# Scale-up of anaerobic 1,3-propanediol production by *Clostridium butyricum* DSP1 from crude glycerol

**DOI:** 10.1186/1471-2180-14-45

**Published:** 2014-02-20

**Authors:** Daria Szymanowska-Powałowska, Wojciech Białas

**Affiliations:** 1 Department of Biotechnology and Food Microbiology, Poznań University of Life Sciences, Wojska Polskiego 48, Poznan, 60-527, Poland

**Keywords:** Batch culture, *Clostridium butyricum*, Glycerol, Fed-batch fermentation, Scale-up, 1,3-Propanediol

## Abstract

**Background:**

As the production of biofuels from raw materials continuously increases, optimization of production processes is necessary. A very important issue is the development of wasteless methods of biodiesel production. One way of utilization of glycerol generated in biodiesel production is its microbial conversion to 1,3-PD (1,3-propanediol).

**Results:**

The study investigated the scale-up of 1,3-PD synthesis from crude glycerol by *Clostridium butyricum*. Batch fermentations were carried out in 6.6 L, 42 L and 150 L bioreactors. It was observed that cultivation of *C. butyricum* on a pilot scale did not decrease the efficiency of 1,3-PD production. The highest concentrations of 1,3-PD, 37 g/L for batch fermentation and 71 g/L for fed-batch fermentation, were obtained in the 6.6 L bioreactor. The kinetic parameters of 1,3-PD synthesis from crude glycerol established for batch fermentation were similar regarding all three bioreactor capacities. During fed-batch fermentation, the concentration of 1,3-PD in the 150 L bioreactor was lower and the substrate was not completely utilized. That suggested the presence of multifunctional environmental stresses in the 150 L bioreactor, which was confirmed by protein analysis.

**Conclusion:**

The values of effectivity parameters for 1,3-PD synthesis in batch fermentations carried out in 6.6 L, 42 L and 150 L bioreactors were similar. The parameters obtained during fed-batch fermentations in the 150 L bioreactor differed in the rate and percentage of substrate utilization. The analysis of cell proteins demonstrated that a number of multifunctional stresses occurred during fed-batch fermentations in the 150 L bioreactor, which suggests the possibility of identifying the key stages in the biochemical process where inhibition of 1,3-PD synthesis pathways can be observed.

## Background

The current tendency to use alternative energy sources has resulted in a significant increase in the production of biofuels that are a wide range of fuels derived from biomass. The world's most common biofuel is biodiesel, made from vegetable oils, animal fats or recycled greases.

The production of biodiesel in the USA alone rose nearly threefold, from 1.561bn tons in 2010 to 4.409bn tons in 2012 [1]. The total production of biodiesel in the 27 states of the European Union in 2010 was over 21 m tons. However, a rise in biodiesel production generates a huge amount of crude glycerol (1 part of glycerol per 10 parts of biodiesel produced) [2]. In the past few years the price of refined glycerol dropped from $1.15 to $0.66 per kilogram and the price of waste glycerol also decreased, from $0.44 to $0.11 per kilogram [3]. Glycerol has the advantage of being a natural and least expensive substrate in the biotechnological process [4]. This hard-to-manage waste product can be used as a component of production media for bacteria that synthesize dihydroxyacetone (*Acetobacter* sp., *Gluconobacter* sp.), butanol (*Clostridium pasteurianum* ATCC® 6013TM), propionic acid (*Propionibacterium*), succinic acid (*Basfia succiniciproducens DD1*), polyhydroxyalkanoates (PHAs) (*Pseudomonas oleovorans* NRRL B-14682), vitamin B12 (*Propionibacterium freudenreichii* ssp. shermanii) or ethanol (a nonpathogenic strain of *Kluyvera cryocrescens* S26) [5-12]. Crude glycerol is also used as a source of carbon for yeasts. It can be used in the growth medium for fodder yeasts or as a substrate for the synthesis of citric acid (*Yarrowia lipolytica* N15), acetic acid, mannitol (*Yarrowia lipolytica* LFMB 19), erythritol (*Yarrowia lipolytica* Wratilsavia K1) and during fat synthesis - single-cell oil (*Yarrowia lipolytica* ACA-DC 50109) [13-16]. Bioglycerol may be successfully used to synthesize fumaric acid (*Rhyzopus* sp.) and arabitol (*Debaryomyces hansenii*), and as a cosubstrate for the synthesis of xylitol (*Candida* sp.) [17,18]. The best solution to utilize glycerol is its microbiological conversion to industrially useful metabolites, such as 1,3-propanediol (1,3-PD), which can be used in many different ways as valuable chemical agents including intermediate applications in organic synthesis, in the production of biodegradable polymers (polyesters, polyethers, polyurethanes), cosmetics, lubricants, medicines as well as in the synthesis of heterocyclic compounds [19,20]. 1,3-PD may be produced chemically or microbiologically [19,21]. At present chemical methods are being replaced by microbiological technologies [21]. In the microbiological conversion of glycerol to 1,3-PD bacteria of the *Clostridium* spp.*, Klebsiella* spp.*, Citrobacter* spp.*,* and *Lactobacillus* spp. are commonly used [19,22,23]. The key problem in the application of 1,3-PD production by bacteria for industrial purposes is the maintenance of lab-scale concentrations of 1,3-PD and other kinetic parameters during industry-scale synthesis [24-28]. The need to apply growth medium sterilization or in-process gas management, especially at a large industrial scale, also affects the cost of the biotechnological process [29-31]. Other challenges are biomass flocculate, foaming, and the adhesion of bacteria to bioreactor walls. Despite the many problems involved in the use of waste substrate in the biotechnological process, there are numerous examples of highly efficient 1,3-PD producing strains that depend exclusively on crude glycerol for the carbon source. The extent of difficulty may be reflected by the limited data on the scale-up of biotechnological processes provided by the literature. Despite the fact that the microbial synthesis of 1,3-PD by the *Clostridium* genus is well documented, very few authors have discussed pilot-scale fermentations [22-24,27,28,32-34].

In this work, a newly isolated *C. butyricum* strain was used to convert crude glycerol to 1,3-PD. The main aim of the research was to investigate the efficiency and other vital parameters of 1,3-PD production in bioreactors of various capacity (6.6 L, 42 L, 150 L) in order to determine the possibility of achieving desired production parameters on a given scale.

## Methods

### Microorganism

In the process of converting crude glycerol to 1,3-PD the bacteria strain *C. butyricum* DSP1 was used. It was previously isolated from ruminal fluid and put in the collection of the Department of Biotechnology and Food Microbiology, Poznan University of Life Sciences, Poland, as wall as deposited at the Polish Collection of Microorganisms (PCM).

### Culture medium

The strain was maintained in Reinforced Clostridial Medium (RCM, Oxoid, UK) in serum bottles at 4°C. Pre-cultures of pure culture inoculum were cultivated in Hungate test tubes in an appropriate cultivation medium (37°C, 18 h).

*Clostridium* bacteria were cultured in a chamber for cultivation of anaerobic microorganisms (Whitley MG500, Don Whitley Scientific, Shipley, United Kingdom), without pH regulation or stirring.

### Fermentation medium

The composition of the fermentation medium was (per liter of deionized water): 0.26 g K_2_HPO_4_; 0.02 g KH_2_PO_4_; 1.23 g (NH_4_)_2_SO_4_; 0.1 g MgSO_4_ × 7H_2_O; 0.01 g CaCl_2_ × 2H_2_O; 0.01 g FeCl_2_ × 7H_2_O and 2.0 g yeast extract. The fermentation medium was supplemented with crude glycerol (Wratislavia-Bio, Wroclaw, Poland) at a concentration of 70.0 ± 1.0 g/L in batch fermentation, and 50 g/L ± 1.0 g/L in fed-batch fermentation. The crude glycerol composition was (w/w) 85.6% glycerol, 6% NaCl, 11.2% moisture, and pH 6.5. The media were autoclaved (121°C, 20 min.).

### Fermentation experiments

The batch experiments were performed at three reactor scales, 6.6 L, 42 L (Sartorius Stedim, Germany) and 150 L (BIOFLO III, New Brunswick Sci. Edison, N.J., USA). All bioreactors were equipped with controls for temperature, pH, agitation speed and aeration rate. The pH was controlled at 7.0 by automatic addition of 1 M NaOH and all fermentation experiments were carried out at 37°C. In the 6.6 L and 42 L bioreactors the anaerobic conditions were sustained by continuous nitrogen sparging at a flow rate of 0.1 vvm whereas in the 150 L bioreactor the medium was sparged with N_2_ for 3 h before and for 1 h after inoculation. As the fermentation process progressed, the medium was sparged with N_2_ for 30 min. once every 24 h. All the bioreactors were inoculated with 10% (v/v) of the pre-inoculum cultures.

The fed-batch experiments were performed at two reactor scales, in 6.6 L and 150 L fermenters. The fermentation was carried out at 5% of the initial glycerol concentration. The major dimensions of the bioreactors used in this study are presented in Table 1. The following equations were used to calculate the main fermentations parameters:

**Table 1:** Stirred-tank reactor characteristics

**Dimension/operating condition**	**Scale**		
Nominal volume, V (L)	6.6	42	150
Working volume, VL (m^3^)	0.005	0.030	0.120
Impeller tip speed, TS (m/s)	0.20096	0.20096	0.20096
Agitation speed, N (rmp)	60.00	36.57	26.50
Number of impeller	2	3	3
Impeller type	Rushton	Rushton	Rushton
Liquid height, HL (m)	0.25	0.46	0.72
Impeller diameter, DI (m)	0.064	0.105	0.150
Reactor diameter, DT (m)	0.16	0.29	0.45
Reactor hight, HT (m)	0.34	0.63	0.98
HT/DT	2.12	2.17	2.18
DI/DT	0.40	0.36	0.33
Inoculum volume, Vx (10%) (m^3^)	0.0005	0.0030	0.0114

Impeller tip speed (ITS):

(1) $${\mathrm{ITS}}_{m/s}=\prod {\mathrm{ND}}_{I}/60$$

where π = 3.142

N = Agitation speed

D_I_ = Impeller diameter

Agitation speed (N):

(2) $$N\;\left(\mathrm{rpm}\right)=60\;\mathrm{ITS}/D_{I}\prod$$

Inoculum volume (V_x_):

(3) $$V_{x}=0.1V_{L}$$

### Analytical methods

The 1,3-PD, glycerol and organic acids were assayed by high-performance liquid chromatography. Samples for chemical analysis were first centrifuged at 10,000 *g* for 10 min at 4°C (Multifuge 3SR, Germany), filtered through a 0.22 μm membrane filter (Millex-GS, Millipore, USA), and then analyzed on an HPLC system (Agilent Technologies 1200 series).

An Agilent Technolgies 1200 series system equipped with a refractive index detector was used. Analyses were performed isocratically at a flow rate of 0.6 mL/min on an Aminex HPX-87H 300 × 7.8 column (Bio-Rad, CA, USA) at a constant temperature of 65°C. H_2_SO_4_ (0.5 mN) was the mobile phase. External standards were applied for identification and quantification of peak areas. Retention times (Rt) determined for the target compounds were as follows: 1,3-PD - 17.17 min; glycerol - 13.03 min; butyric acid - 20.57 min; acetic acid - 14.4 min and lactic acid - 11.19 min.

### Protein analyses

Proteins were reduced (10 mM DTT, 30 min, 56°C) and alkylated with iodoacetamide in darkness (45 min, 20°C) and digested overnight with 10 ng/μL trypsin. The resulting peptide

mixtures were applied to the RP-18 pre-column of a UPLC system (Waters) using water containing 0.1% FA as a mobile phase and then transferred to a nano-HPLC RP-18 column (internal diameter 75 μm, Waters) using ACN gradient (0 – 35% ACN in 160 min) in the presence of 0.1% FA at a flow rate of 250 μL/min. The column outlet was coupled directly to the ion source of an Orbitrap Velos mass spectrometer (Thermo). Each sample was measured in duplicate - once for protein sequencing (data-dependent MS to MS/MS switch) and once for quantitative information (MS only, sequencing disabled).

The acquired MS/MS data were pre-processed with Mascot Distiller software (v. 2.3, MatrixScience) and a search was performed with the Mascot Search Engine MatrixScience, Mascot Server 2.4) against the set of *Clostridium* protein sequences derived from Uniprot, merged with its randomized version (16294 sequences; 5095802 residues).

The proteins that exactly matched the same set of peptides were combined into a single cluster. The mass calibration and data filtering were carried out with MScan software.

The lists of peptides that matched the acceptance criteria from the LC-MS/MS runs were merged into one common list. This common list was overlaid onto 2-D heat maps generated from the LCMS profile datasets by tagging the peptide-related isotopic envelopes with corresponding peptide sequence tags on the basis of the measured/theoretical mass difference, the deviation from the predicted elution time, and the match between the theoretical and observed isotopic envelopes. The abundance of each peptide was determined as the height of a 2-D fit to the monoisotopic peak of the tagged isotopic envelope. Quantitative values were normalized with LOWESS, proteins with more than 80% common peptides were clustered and the peptides unique for the cluster were used for statistical analysis. Only proteins with q-value below 0.05 or those present in only one of two compared analytical groups were taken into consideration during further analysis. The protein concentration was measured by Bradford’s method [35].

## Results and discussion

### Batch fermentation

Microbiological synthesis of 1,3-PD by *C. butyricum* DSP1 was carried out at an increasing capacity of bioreactors. The efficiency of 1,3-PD production from crude glycerol during the scale-up process was investigated. For this purpose batch fermentations were performed in 6.6 L, 42 L and 150 L bioreactors. The results obtained were used to calculate the basic kinetic parameters of the fermentation processes (Table 2). It was found that the scale-up process did not have any effect on the growth of microorganisms or 1,3-PD synthesis.

**Table 2:** **Kinetic parameter values from *C. butyricum *** **DSP1 in 6.6 L, 42 L and 150 L bioreactors**

**Parameter/fermentation scale**	**6.6 L**	**42 L**	**150 L**
Time of fermentation (h)	33	32	28
Max biomass, X_max_ (g/L)	1.2	1.2	1.3
Time taken to reach max biomass, t (h)	15	16	14
Max specific growth rate, μ (1/h)	0.067	0.062	0.071
Max 1,3-PD concentration, 1,3-PD_max_ (g/L)	37.63 ± 1.2	36.40 ± 1.6	37.20 ± 1.4
1,3-PD productivity P_1,3-PD_ (g/L/h)	1.12	1.13	1.33
1,3-PD yield, Y_1,3-PD_ (g_1,3- PD_/g_Gly_)	0.53	0.52	0.53
Max butyric acid concentration, But_max_ (g/L)	4.26 ± 0.09	3.57 ± 0.08	4.22 ± 0.07
Butyric acid productivity P_But_ (g/L/h)	0.13	0.11	0.15
Butyric acid yield, Y_But_ (g_But_/g_Gly_)	0.06	0.05	0.06
Max acetic acid concentration, Ace_max_ (g/L)	2.0 ± 0.03	1.9 ± 0.03	2.2 ± 0.02
Acetic acid productivity P_Ace_ (g/L/h)	0.06	0.06	0.08
Acetic acid yield, Y_Ace_ (g_Lac_/g_Gly_)	0.03	0.03	0.03
Max lactic acid concentration, Lac_max_ (g/L)	3.14 ± 0.02	2.84 ± 0.03	3.63 ± 0.04
Lactic acid productivity P_Lac_ (g/L/h)	0.09	0.09	0.12
Lactic acid yield, Y_Lac_ (g_Lac_/g_Gly_)	0.04	0.04	0.05

Each point is the mean value of two independent measurements.

The concentration of the diol in the 150 L bioreactor was close to concentrations achieved in the 6.6 L and 42 L bioreactors and averaged 37 g/L. In all batch fermentations the glycerol was completely utilized. However, some differences were observed in the productivity of the bioreactors as their capacity increased, with the 150 L bioreactor giving 1.33 g/L/h, which probably depended on the quantity of biomass (Table 2). The plateau of microorganism growth was achieved in the14th hour of cultivation and was followed by the stationary phase. The profiles of by-products formed in the respective bioreactors were comparable.

The first scale-up experiments on 1,3-PD synthesis from glycerol (by *C. butyricum* DSM 5431) were described by Günzel et al. [24] and involved fermentation starting in a 1.4 L bioreactor and proceeding to a 2000 L bioreactor. The highest concentrations of 1,3-PD were obtained in 30 L and 1200 L air-lift bioreactors. Regarding the stirred-tank bioreactors used in that study (based on the same working principle as those used during the experiments described in our paper) the maximal level of 1,3-PD, 56 g/L, was observed in the 30 L bioreactor. However, Günzel et al. [24] did not use crude but pure glycerol as a carbon source. Papanikolaou et al. [36] studied 1,3-PD synthesis from glycerol by *C. butyricum* F2b in batch fermentation and received a final 1,3-PD concentration of 47.1 g/L from 65 percent pure glycerol. The yield of the process was 0.53 g/g, equal to that achieved in the present work. Anand and Saxena [37] while testing *Citrobacter freundii* obtained a yield level of 0.51 g/g for 1,3-PD synthesis from crude glycerol and a final 1,3-PD concentration of 25.6 g/L.

### Fed-batch fermentation

**Figure 1:**
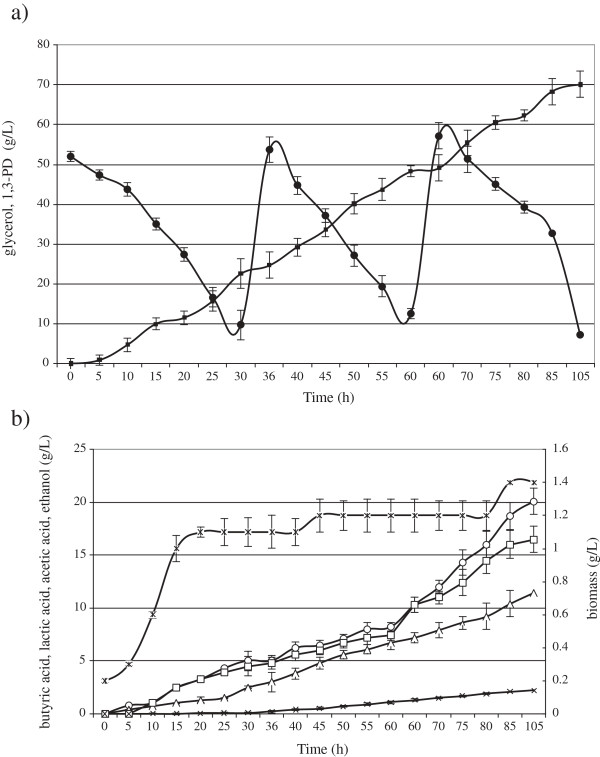
**Kinetics of glycerol consumption (filled circles) and 1,3-propanediol production (filled squares) (a); butyric acid (open circles), lactic acid (open squares), acetic acid (open triangles), ethanol (cross), production and biomass growth (stars) (b) during growth of *C. butyricum *** **DSP1 in fed-batch in 6.6 L bioreactor experiments.**

**Figure 2:**
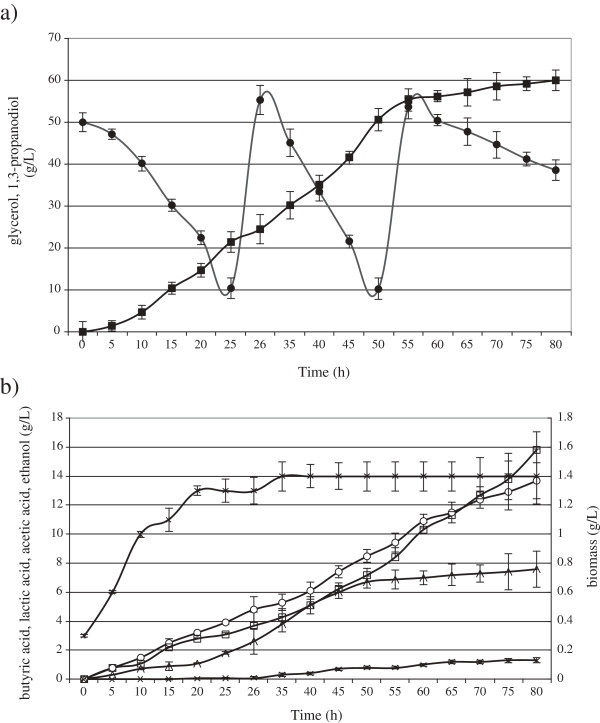
**Kinetics of glycerol consumption (filled circles) and 1,3-propanodiol production (filled squares) (a); butyric acid (open circles), lactic acid (open squares), acetic acid (open triangles), ethanol (cross), production and biomass growth (stars) (b) during growth of *C. butyricum *** **DSP1 in fed-batch in 150 L bioreactor experiments.**

The batch fermentations were carried out to check whether the optimization of the cultivation medium and the fermentation tests were properly conducted on a laboratory scale [38]. The purpose of the fed-batch fermentations was to achieve an increased production of 1,3-PD. This method enables the use of high glycerol amounts and allows for the reduction of stresses resulting from the high osmolality of production media [30]. The kinetics of 1,3-PD production in fed-batch fermentation was compared between the 6.6 L and the 150 L bioreactors (Figure 1 and Figure 2). The concentration of glycerol at the start of fermentation was 50 g/L. The highest concentration of 1,3-PD, 71 g/L, was obtained in the 6.6 L bioreactor from 132 g/L glycerol (Figure 1a). In the 150 L bioreactor the final product concentration did not exceed 60 g/L (Figure 2a).

In the beginning the basic kinetic parameters of batch and fed-batch fermentations were comparable, with the only difference in the length of the adaptive phase of bacteria growth. As a result, the stationary phase started as early as 5 hours after inoculation of the fermentation medium. However, the rate of 1,3-PD production significantly decreased after adding the second portion of glycerol and biomass growth was no longer observed. It has been reported that biological processes occurring on a large scale are limited by environmental stresses [22]. 1,3-PD synthesis is known to be influenced by such factors as osmotic stress (connected with high concentrations of raw material), toxic stress (caused by increased concentrations of organic acids and 1,3-PD), mechanical stress (pressure, shear force) and, in the case of anaerobic bacteria, oxidative stress. The incomplete utilization of crude glycerol and the inhibition of 1,3-PD production in fed-batch fermentation in this work resulted probably from the accumulation of toxic by-products generated during 1,3-PD synthesis, such as butyric (14–20 g/L), lactic (16–17 g/L), and acetic (8–11 g/L) acids. Similar findings were presented by Biebl [39], who noted that 19 g/L of butyric acid and 27 g/L of acetic acid inhibited the production of 1,3-PD by *C. butyricum*. Moreover, the addition of new portions of crude glycerol reduced the metabolic activity of the bacteria (Figure 2b) by increasing the osmotic pressure and introducing impurities contained in crude glycerol. That substrate may carry substances inhibiting the growth and metabolism of microorganisms: sodium salts, heavy metal ions, soaps, methanol, and free fatty acids (linolenic, stearic, palmitic, oleic and linoleic) [40,41]. Venkataramanan et al. [41] analyzed the influence of impurities contained in crude glycerol such as methanol, salts and fatty acids on the growth and metabolism of *C. pasteurianum* ATCC 6013, responsible for synthesizing butanol and 1,3-PD. They found that fatty acids (mainly linoleic acid) had the most adverse impact on the utilization of glycerol by *Clostridium* bacteria. These acids have been reported to significantly diminish cell viability [42]. Studies similar to those of Venkataramanan et al. [41] were performed by Chatzifragkou et al. [40]. When oleic acid was added to the growth medium at 2% (w/w of glycerol), a total preclusion of the strain was observed. In order to investigate whether the nature of oleic acid itself or the presence of the double bond induced inhibition, stearic acid was added into the medium at the same concentration (2%, w/w, of glycerol). No inhibitory effect was observed, suggesting that the presence of the double bond played a key role in the growth of the microorganisms. Also salts are considered to be toxic components of crude glycerol [40,41]. Monovalent salts have been shown to negatively affect the cell membrane by reducing the van der Waals forces between the lipid tails within it [43].

In this work glycerol contained 0.6 g/L of sodium chloride. The concentration of sodium ions increased during fed-batch fermentation as the second portion of contaminated glycerol was added. That did not carry any complex nutrients, which probably further limited the metabolic activity of the bacteria and caused incomplete substrate utilization. Similar observations were made by Dietz and Zeng [44]. Hirschmann et al. [45] achieved a concentration of 100 g/L with the use of *Clostridium* but the feeding contained 40 g/L yeast extract apart from crude glycerol. Additionally, NaOH was used to regulate pH. Growth of *C. butyricum* was inhibited at 12 g/L sodium ions [46]. Table 3 presents results of this study as compared to those of other authors. It is possible that another stress factor was the insufficient transfer of gas (N_2_) in the bioreactor leading to oxidative stress and, probably, to the inactivation of the oxygen-sensitive enzyme NADH-ferredoxin reductase, causing the change observed in the ratio of lactate to butyrate in the 150 L bioreactor (Figure 2b). Although during 1,3-PD synthesis from glycerol by *C. butyricum* butyric, acetic and lactic acids as well as ethanol are produced, the main byproducts of a proper conversion of glycerol to 1,3-PD are butyrate and acetate. An increased content of lactic acid indicates that the process is blocked probably due to substrate excess, a high concentration of toxic carbon monoxide or stoppage at the stage of pyruvate generation. Chatzifragkou et al. [27] found an increase in the activity of lactate dehydrogenase in a 1 L bioreactor at a high substrate concentration in the absence of continuous N_2_ sparging.

**Table 3:** The most promising bacteria strains capable of efficient 1,3-PD synthesis from crude glycerol

**Strain**	**Fermentation method**	**C**_**1,3-PD **_**[g/L]**	**Y**_**1,3-PD **_**[g**_**1,3-PD**_**/g**_**Gly**_**]**	**Crude glycerol purity (% w/w)**	**Ref.**
*C. butyricum* AKR102a	Fed-batch	76.2	0.51	55	[28]
*C. butyricum* VPI 1718	Fed-batch	67.9	0.55	81.0	[29]
*Clostridium sp.*	Fed-batch	80.1	0.56	ND	[28]
*C. butyricum* DSP1	Fed-batch	71.0	0.54	85.6	Present study
*K. pneumoniae* DSM 4799	Fed-batch	80.2	0.45	80.0	[47]
*K. pneumoniae* DSM 2026	Fed-batch	53.0	ND	85.0	[48]
*K. oxytoca* FMCC-197	Fed-batch	50.1	0.40	81.0	[31]
*C. freundii* FMMC-B 294 (VK-19)	Fed-batch	68.1	0.40	81.0	[30]
Mix culture	Fed-batch	70.0	0.47	81.0	[44]

ND – non-designated, C_1,3-PD –_ maximal final 1,3-PD concentration obtained, Y_1,3-PD_ – maximal yield of glycerol conversion to 1,3-PD obtained.

The effect was more pronounced in large-scale fermentations than in small-scale processes and depended on the vessel geometry. Some studies have shown that nitrogen sparging throughout fermentation has a positive effect on the process carried out with *C. butyricum* as it influences bacteria metabolism because of the expulsion of dissolved CO_2_[34]. In the experiments of Chatzifragkou et al. [27] continuous sparging with N_2_ allowed for an increased 1,3-PD yield and biomass formation that correlated with a decreased production of lactic acid. Metsoviti et al. [31] observed quite a different effect. Continuous sparging of the fermentation medium with nitrogen during fermentation induced by *K. oxytoca* produced a shift in the metabolism of glycerol towards ethanol whereas non-sparging favored 1,3-PD synthesis. Moreover, 1,3-PD also had an inhibiting impact on the process of fermentation. The inhibiting influence of 1,3-PD on the metabolic activities of bacteria has been described by many authors and its concentration was found toxic at a level of 60–90 g/L [39,49-51]. Colin et al. [51] described the influence of glycerol and 1,3-PD concentrations on the growth of *C. butyricum* CNCM 1211. It was demonstrated that the strain exhibited resistance to 1,3-PD up to a concentration of 60 g/L. Papanikolaou et al. [52] showed the resistance of *C. butyricum* to the concentration of 1,3-PD not exceeding 80 g/L. Ringel et al. [53] isolated two strains of *C. butyricum* (AKR91b and AKR102a) able to grow and synthesize 1,3-PD in a medium supplemented with 1,3-PD at its initial concentration of 60 g/L. The limiting concentration of 1,3-PD was 77 g/L for another isolate (AKR92a). Both glycerol and 1,3-PD have been observed to cause osmotic stress [4]. In batch processes, the osmolality of fermentation wort is constant (1,3-PD concentration goes up while glycerol concentration falls). In fed-batch fermentation, the ratio of glycerol to 1,3-PD tends to vary. The osmotic pressure rises as a result of 1,3-PD accumulation and addition of new portions of glycerol. The problem of increasing osmotic pressure may be solved by replacing fed-batch fermentation with continuous fermentation. It has been observed that an elevated alcohol (ethanol, butanol, methanol) concentration may also negatively influence microorganisms involved in fermentation [54]. The metabolites formed during 1,3-PD synthesis from glycerol by *Clostridium* bacteria include ethanol and butanol. As proposed by Shimizu and Katsura [55], alcohols are responsible for the inhibition of the membrane ATPase and transport mechanisms. Bowles and Ellefson [56] as well as Gottwald and Gottschalk [57] pointed to the uncoupling role of alcohols through suppression of the transmembranary pH gradient. In *C. acetobutylicum*, high concentrations of butanol inhibit active nutrient transport the membrane-bound ATPase and glucose uptake, partially or completely neutralizing the membrane ΔpH [57]. In the present study, the maximum ethanol concentration during fed-batch fermentation in the 150 L bioreactor was 2.2 g/L (Figure 2b). That alcohol was possibly another factor adding to the environmental stresses acting on the microorganisms. Venkataramanan et al. [41] examined the influence of methanol on the viability and metabolism of *C. pasteurianum* ATCC™ 6013 and found that the concentration of methanol in the range 2.5-5.0 g/L did not have a negative effect on the production of the main metabolite.

A vital yet costly stage of biotechnological processes based on the use of microorganisms is sterilization of growth media and technological apparatus. Elimination of that stage, especially from industrial-scale processes, could reduce costs and lower the price of the final product. Successful non-sterile fermentations have been performed during the synthesis of 1,3-PD from glycerol [29-31,44]. Chatzifragkou et al. [29] presented results of fed-batch fermentation showing a nearly negligible difference of 1.6 g/L for 1,3-PD concentrations obtained under non-sterile and sterile conditions. Similarly promising findings were made in non-sterile fermentation experiments involving *K. oxytoca* FMCC-197 [31]. A productivity study by Dietz and Zeng [44] on the non-sterile fermentation of crude glycerol with the use of inocula received from three biogasworks demonstrated an increase in the synthesis of the main product even above the level of theoretical productivity. That was probably caused by the presence of strains able to metabolize glycerol other than *C. butyricum* and the introduction of an additional carbon source that was contained in the consortium.

### Analysis of some protein markers of environmental stresses

The development of bioprocess technology has led to a greater production of metabolites, especially on an industrial scale. Large-scale production is connected with several problems such as the need to ensure optimal temperature and osmotic pressure as well as a non-inhibiting level of metabolites and to provide proper nutrients, and the fact that bacteria cells are prone to mechanical damage caused by shear force.

In this study, in order to determine the environmental stresses resulting from the addition of glycerol in fed-batch fermentation some cell proteins considered to be stress markers were analyzed (Table 4).

**Table 4:** **Proteomic analysis of stress response in *C. butyricum *** **DSP1**

**Protein names**	**Gene/ORF names**	**Number ID**	**Mass (Da)**	q-value*	Fold change**	Fold change***
HSP20	CLP_1581	C4ILE7	17.07	0.0024	1.62	3.41
GroEL (HSP60)	groL	B1R088	57.90	0.0056	2.14	5.31
DnaK (HSP70)	dnak	C4IDG2	65.64	0.0165	1.32	3.72
HSP90	CLP_0987	C4IJL7	75.22	0.0076	0.23	0.31
SpoOA	Spo0A	B1QU80	31.45	0.0021	1.32	3.72

*q-value – statistical significance of obtained results.

**fold change between samples from batch and fed-batch fermentation – after adding the first portion of glycerol (26th hour).

***fold change between samples from batch and fed-batch fermentation – after adding the second portion of glycerol (52nd hour).

The differences between the level of the heat shock proteins HSP20, HSP60 (GroEL), HSP70 (DnaK), HSP90 and the transcription factors of sporulation process of SpoOA were observed. The literature points to Hsp60 (GroEL) as a protein associated with the response of the genus *Clostridium* to osmotic, toxic and temperature stresses [58,59].

Hennequin et al. [59] observed the influence of increased temperature (30-48°C) on the level of GroEL in *C. difficile* and found that after incubation at 43°C the level of this protein was 3 times greater than at 30°C. For *C. acetobutylicum*, a rise in the temperature from 30 to 42°C resulted in the appearance of 15 heat shock proteins belonging to the family HSP60 and HSP70 [60].

In the current work, heat shock proteins were detected in metabolically active cells able to synthesize 1,3-PD in batch and fed-batch fermentations. During batch fermentation the levels of all proteins studied were low whereas in fed-batch fermentation the amount of HSP60 increased twofold and of HSP20 1.5 times after adding the first portion of crude glycerol.

After adding the second portion of glycerol, the level of both proteins rose twofold (Table 4). Under conditions of environmental stress, the protein HSP20 prevents undesirable interactions between proteins and is a transduction signal. The function of HSP60 is to coat molecules of other proteins preventing their denaturation [59]. By contrast, the level of HSP90 (heat shock marker) was constant, which may be explained by the fact that temperature stress did not occur in the fed-batch process. In the 150 L bioreactor, following the addition of the first and second portions of glycerol, an increase of the transcription factor SpoOA, responsible for synthesizing GroEL, GroES and HSP18 heat shock proteins, was observed [61]. The synthesis of heat shock proteins is probably connected with sporulation in *Clostridium* spp. [58,62]. In the present work, despite the fact that stress proteins were identified in fed-batch fermentation, the level of enzymes taking part in 1,3-PD synthesis, glycerol dehydratase and 1,3-PD dehydrogenase, did not change. Since the response of cells to multifunctional stresses requires an additional amount of energy to trigger a cascade of biochemical reactions, the metabolic activity of cells is reduced and so the production of the target metabolite is diminished.

## Conclusions

This study analyzed changes in the kinetics of 1,3-PD synthesis from crude glycerol during a scale-up process. The values of effectivity parameters for 1,3-PD synthesis in batch fermentations carried out in 6.6 L, 42 L and 150 L bioreactors were similar. The parameters obtained during fed-batch fermentations in the 150 L bioreactor differed in the rate and percentage of substrate utilization. The analysis of cell proteins demonstrated that a number of multifunctional stresses occurred during fed-batch fermentations in the 150 L bioreactor, which suggests the possibility of identifying the key stages in the biochemical process where inhibition of 1,3-PD synthesis pathways can be observed. Based on the knowledge of mechanisms underlying those critical phases it may be possible to change synthesis pathways at the molecular level by, for example, over-expression or knock-out of genes in order to modify the microorganisms involved in synthesis in terms of their biotechnological potential and resistance to environmental stresses.

## Competing interests

The authors declare that they have no competing interests.

## Authors’ contributions

Conceived and designed the experiments: DSP, WB. Performed the experiments: DSP Analyzed the data: DSP. Contributed reagents/materials/analysis tools: DSP, WB. Wrote the paper: DSP. Both authors read and approved the final manuscript.

## References

[B1] Monthly Biodiesel Production Report U.S. Energy Information Administration 2013 Washington, DC 20585, USA

[B2] Abad S Turon X Vaporization of biodiesel derived glycerol as a carbon source to obtain added-value metabolites: Focus on polyunsaturated fatty acids Biotechnol Adv 2012 30 733 741 10.1016/j.biotechadv.2012.01.002 22261015

[B3] Yang FX Hanna MA Sun RC Value-added uses for crude glycerol - A byproduct of biodiesel production Biotechnol Biofuels 2012 5 13 10.1186/1754-6834-5-13 22413907 PMC3313861

[B4] Kośmider A Leja K Czaczyk K Montero G, Stoytcheva M Improved utilization of crude glycerol by-product from biodiesel production Biodiesel-quality, emissions and by-products 2011 Coratia: InTech 341 578

[B5] Nabe K Izuo N Yamada S Chibata I Conversion of glycerol to dihydroxyacetone by immobilized whole cells of *Acetobacter xylinum* Appl Env Microbiol 1979 38 1056 1060 16345471 10.1128/aem.38.6.1056-1060.1979 PMC291244

[B6] Claret C Salmon JM Romieu C Bories A Physiology of *Gluconobacter oxydans* during dihydroxyacetone production from glycerol Appl Microbiol Biotechnol 1994 41 359 365 10.1007/BF00221232

[B7] Bories A Himmi E Jauregui JJA Pelayo-Ortiz C Gonzales VA Glycerol fermentation with Propionibacteria and optimization of the production of propionic acid Sci Aliment 2004 24 21 135

[B8] Taconi KA Venkataramanan KP Johnson DT Growth and solvent production by *Clostridium pasteurianum* ATCC® 6013™ utilizing biodiesel-derived crude glycerol as the sole carbon source Environ Prog Sustain Energy 2009 28 100 110 10.1002/ep.10350

[B9] Scholten E Renz T Thomas J Continuous cultivation approach for fermentative succinic acid production from crude glycerol by *Basfia succiniciproducen* DD1 Biotechnol Lett 2009 31 1947 1951 10.1007/s10529-009-0104-4 19705071

[B10] Ashby RD Solaiman DKY Strahan GD Efficient utilization of crude glycerol as fermentation substrate in the synthesis of poly (3-hydroxybutyrate) biopolymers J Am Oil Chem Soc 2011 88 949 959 10.1007/s11746-011-1755-6

[B11] Choi WJ Hartono MR Chan WH Yeo SS Ethanol production from biodiesel-derived crude glycerol by newly isolated *Kluyvera cryocrescen* Appl Microbiol Biotechnol 2011 89 1255 1264 10.1007/s00253-010-3076-3 21212944

[B12] Kośmider A Białas W Kubiak P Drożdżyńska A Czaczyk K Vitamin B12 production from crude glycerol by *Propionibacterium freudenreichii* ssp. shermanii: optimization of medium composition through statistical experimental designs Bioresour Technol 2012 105 128 133 22178491 10.1016/j.biortech.2011.11.074

[B13] Rymowicz W Rywińska A Marcinkiewicz M High-yield production of erythritol from raw glycerol in fed-batch cultures of *Yarrowia lipolytica* Biotechnol Lett 2009 31 377 380 10.1007/s10529-008-9884-1 19037599

[B14] Kamzolova SV Fatykhova AR Dedyukhina EG Anastassiadis SG Golovchenko NP Morgunov IG Citric acid production by yeast grown on glycerol-containing waste from biodiesel industry Food Technol Biotechnol 2011 49 65 74

[B15] Chatzifragkou A Makri A Belka A Bellou S Mayrou M Mastridou M Mystrioti P Onjaro G Aggelis G Papanikolaou S Biotechnological conversion of biodiesel derived waste glycerol by yeast and fungal species Energy 2012 36 1097 1108

[B16] Papanikolaou S Aggelis G Biotechnological valorization of biodiesel derived glycerol waste through production of single cell oil and citric acid by *Yarrowia lipolytica* Lipid Technol 2009 21 83 87 10.1002/lite.200900017

[B17] Moon SK Wee YJ Yun JS Ryu HW Production of fumaric acid using rice bran and subsequent conversion to succinic acid through a two-step process Appl Biochem Biotechnol 2004 115 843 856 10.1385/ABAB:115:1-3:0843 15054237

[B18] Arruda PV Felipe MG Role of glycerol addition on xylose-to-xylitol bioconversion by *Candida guilliermondii* Curr Microbiol 2008 58 274 278 19034573 10.1007/s00284-008-9321-7

[B19] Amaral PFF Ferreira TF Fontes GC Coelho MAZ Glycerol valorization: new biotechnological routes Food Bioprod Proc 2009 87 179 186 10.1016/j.fbp.2009.03.008

[B20] Koganti S Kuo TM Kurtzman CP Production of arabitol from glycerol: strain screening and study of factors affecting production yield Appl Microbiol Cell Physiol 2011 90 257 267 10.1007/s00253-010-3015-3 21127857

[B21] Zeng AP Biebl H Bulk chemicals from biotechnology: the case of 1,3-propanediol production and the new trends Adv Biochem Eng/Biotechnol 2002 74 239 259 10.1007/3-540-45736-4_11 11991182

[B22] Kubiak P Leja K Myszka K Celińska E Spychała M Szymanowska-Powałowska D Czaczyk K Grajek W Physiological predisposition of various *Clostridium* species to synthetize 1,3-propanediol from glycerol Proc Biochem 2012 47 1308 1319 10.1016/j.procbio.2012.05.012

[B23] Metsoviti M Paramithiotis S Drosinos EH Galiotou-Panayotou M Nychas GJE Zeng AP Papanikolaou S Screening of bacterial strains capable of converting biodiesel-derived raw glycerol into 1,3-propanediol, 2,3-butanediol and ethanol Eng Life Sci 2012 12 57 68 10.1002/elsc.201100058

[B24] Günzel B Yonsel S Deckwer WD Fermentative production of 1,3-propanediol from glycerol by *Clostridium butyricum* up to a scale of 2 m^3^ Appl Microbiol Biotechnol 1991 36 289 294

[B25] Liu HJ Zhang DJ Xu YH Mu S YQ Xiu ZL Microbial production of 1,3-propanediol from glycerol by *Klebsiella pneumoniae* under micro-aerobic conditions up to a pilot scale Biotechnol Lett 2007 29 1281 1285 10.1007/s10529-007-9398-2 17503001

[B26] Zheng ZM Guo NN Hao J Cheng KK Sun Y Liu DH Scale-up of micro-aerobic 1,3-propanediol production with *Klebsiella pneumonia* CGMCC 1.6366 Proc Biochem 2009 44 944 948 10.1016/j.procbio.2009.04.017

[B27] Chatzifragkou A Aggelis G Komaitis M Zeng AP Papanikolaou S Impact of anaerobiosis strategy and bioreactor geometry on the biochemical response of *Clostridium butyricum* VPI 1718 during 1,3-propanediol fermentation Bioresour Technol 2011 102 10625 10632 10.1016/j.biortech.2011.09.023 21967709

[B28] Wilkens E Ringel AK Hortig D Willke T Vorlop KD High-level production of 1,3-propanediol from crude glycerol by *Clostridium butyricum* AKR102a Appl Microbiol Biotechnol 2012 93 1057 1063 10.1007/s00253-011-3595-6 21972131

[B29] Chatzifragkou A Papanikolau S Dietz D Doulgeraki AI Nychas GJE Zeng AP Production of 1,3-propanediol by *Clostridium butyricum* growing on biodiesel-derived crude glycerol through a non-sterilized fermentation process Appl Microbiol Biotechnol 2011 91 101 112 10.1007/s00253-011-3247-x 21484206

[B30] Metsoviti M Zeng AP Koutinas AA Papanikolaou S Enhanced 1,3-propanediol production by newly isolated *Citobacter freundii* strain cultivated on biodiesel-derived waste glycerol trough sterile and non-sterile bioprocesses J Biotechnol 2013 163 408 418 10.1016/j.jbiotec.2012.11.018 23220217

[B31] Metsoviti M Paraskevaidi K Koutinas AA Zeng AP Papanikolaou Production of 1,3-propanediol, 2,3-butanediol and ethanol by a newly isolated *Klebsiella oxytoca* strain growing on biodiesel-derived glycerol based media Proc Biochem 2012 47 1872 1882 10.1016/j.procbio.2012.06.011

[B32] Biebl H Menzel K Zeng AP Deckwer WD Microbial production of 1,3-propanediol Appl Microbiol Biotechnol 1999 52 289 297 10.1007/s002530051523 10531640

[B33] González-Pajuelo M Andrade JC Vasconcelos I Production of 1,3- propanediol by *Clostridium butyricum* VPI 3266 using a synthetic medium and raw glycerol J Ind Microbiol Biotechnol 2004 31 442 446 10.1007/s10295-004-0168-z 15378388

[B34] Biebl H Marten S Hippe H Deckwer WD Glycerol conversion to 1,3-propanediol by newly isolated clostridia Appl Microbiol Biotechnol 1992 36 592 597

[B35] Bradford MM Rapid and sensitive method for the quantitation of microgram quantities of protein utilizing the principle of protein-dye binding Anal Biochem 1976 72 248 254 10.1016/0003-2697(76)90527-3 942051

[B36] Papanikolaou S Fakas S Fick M Chevalot I Galiotou-Panayotou M Komaitis M Marc I Aggelis G Biotechnological valorisation of raw glycerol discharged after bio-diesel (fatty acid methyl esters) manufacturing process: production of 1,3-propanediol, citric acid and single cell oil Biomass Bioenergy 2008 32 60 71 10.1016/j.biombioe.2007.06.007

[B37] Anand P Saxena RK A comparative study of solvent-assisted pretreatment of biodiesel derived crude glycerol on growth and 1,3-propanediol production from *Citrobacter freundii* New Biotechnol 2012 29 199 205 10.1016/j.nbt.2011.05.010 21689798

[B38] Szymanowska-Powałowska D Drożdżyńska A Remszel N Isolation of new strains of bacteria able to synthesize 1,3-propanediol from glycerol Adv Microbiol 2013 3 171 180 10.4236/aim.2013.32027

[B39] Biebl H Glycerol fermentation of 1,3-propanediol by *Clostridium butyricum*. Measurement of product inhibition by use of a pH-auxostat Appl Microbiol Biotechnol 1991 35 701 705

[B40] Chatzifragkou A Dietz D Komaitis M Zeng AP Papanikolau S Effect of biodiesel-derived waste glycerol impurities on biomass and 1,3-propanediol production of *Clostridium butyricum* VPI 1718 Biotechnol Bioeng 2010 107 76 84 10.1002/bit.22767 20506102

[B41] Venkataramanan KP Boatman JJ Kurniawan Y Taconi KA Bothun GD Scholz C Impact of impurities in biodiesel-derived crude glycerol on the fermentation by *Clostridum pasteurianum* ATCC 6013 Bioenergy Biofuels 2012 93 1325 1335 10.1007/s00253-011-3766-5 22202963

[B42] Furusawa H Koyama N Effect of fatty acids on the membrane potential of an alkaliphilic *Bacillus* Curr Microbiol 2004 48 196 198 10.1007/s00284-003-4161-y 15057464

[B43] Petrache HI Tristram-Nagle S Harries D Kucerka N Nagle JF Swelling of phospholipids by monovalent salt J Lipid Res 2006 47 302 309 16267342 10.1194/jlr.M500401-JLR200 PMC2689361

[B44] Dietz D Zeng AP Efficient production of 1,3–propanediol from fermentation of crude glycerol with mixed cultures in a simple medium Bioprocess Biosyst Eng 2013 doi:10.1007/s00449-013-0989-0 10.1007/s00449-013-0989-0 23749235

[B45] Hirschmann S Baganz K Koschik I Vorlop KD Development of an integrated bioconversion process for the production of 1,3-propanediol from raw glycerol waters Landbauforschung Völkenrode 2005 55 261 267

[B46] Homann T Tag C Biebl H Deckwer WD Schink B Fermentation of glycerol to 1,3-propanediol by *Klebsiella* and *Citrobacter* strains Appl Microbiol Biotechnol 1990 33 121 126

[B47] Jun SA Moon C Kang CH Kong SW Sang BI Um Y Microbial fed-batch production of 1,3-propanodiol using raw glycerol with suspend and immobilized *Klebsiella pneumoniae* Appl Biochem Biotechnol 2010 161 491 501 10.1007/s12010-009-8839-x 19921491

[B48] Mu Y Teng H Zhang DJ Wang W Xiu ZL Microbial production of 1,3-propanediol by *Klebsiella pneumoniae* using crude glycerol from biodiesel preparation Biotechnol Lett 2006 28 1755 1759 10.1007/s10529-006-9154-z 16900328

[B49] Zeng AP Ross A Biebl H Tag C Günzel B Deckwer WD Multiple product inhibition and growth modeling of *Clostridium butyricum* and *Klebsiella pneumoniae* in glycerol fermentation Biotechnol Bioeng 1994 44 902 911 10.1002/bit.260440806 18618908

[B50] Saint-Amans S Perlot P Goma G Soucaille P High production of 1,3-propanediol from glycerol by *Clostridium butyricum* VPI 3266 in a simply controlled fed-batch system Biotechnol Lett 1994 16 831 836 10.1007/BF00133962

[B51] Colin T Bories A Moulin G Inhibition of *Clostridium butyricum* by 1,3-propanediol and diols during glycerol fermentation Appl Microbiol Biotechnol 2000 54 201 205 10.1007/s002530000365 10968633

[B52] Papanikolaou S Ruiz-Sanchez P Pariset B Blanchard F Fick M High production of 1,3-propanediol from industrial glycerol by a newly isolated *Clostridium butyricum* strain J Biotechnol 2000 77 191 208 10.1016/S0168-1656(99)00217-5 10682279

[B53] Ringel AK Wilkens E Hortig D Willke T Vorlop KD An improved screening method for microorganisms able to convert crude glycerol to 1,3-propanediol and to tolerate high product concentrations Appl Microbiol Biotechnol 2012 93 1049 1056 10.1007/s00253-011-3594-7 21968654

[B54] Nicolaou SA Gaida SM Papoutsakis ET A comparative view of metabolite and substrate stress and tolerance in microbial bioprocessing: From biofuels and chemicals, to biocatalysis and bioremediation Metab Eng 2010 12 307 31 10.1016/j.ymben.2010.03.004 20346409

[B55] Shimizu T Katsura T Steady – state kinetic study o the inhibition of the adenosinetriphosphatase activity of dynein from *Tetrahymena cilia* by glycerol J Biochem 1988 103 99 105 2966149 10.1093/oxfordjournals.jbchem.a122248

[B56] Bowles LK Ellefson WL Effects of butanol on *Clostridium acetobutylicum* Appl Environ Microbiol 1985 50 1165 1170 2868690 10.1128/aem.50.5.1165-1170.1985 PMC238718

[B57] Gottwald M Gottschalk G The internal pH of *Clostridium acetobutylicum* and its effect on the shift from acid to solvent formation Arch Microbiol 1985 143 42 46 10.1007/BF00414766

[B58] Bahl H Müller H Behrens S Joseph H Narberhaus F Expression of heat shock genes in *Clostridium acetobutylicum* FEMS Microbiol Rev 1995 17 341 348 10.1111/j.1574-6976.1995.tb00217.x 7576772

[B59] Gupta SC Sharma A Mishra M Mishra RK Chowdhuri DK Heat shock proteins in toxicology: How close and how far? Life Sci 2010 86 377 384 10.1016/j.lfs.2009.12.015 20060844

[B60] Hennequin C Porcheray F Waligora-Dupriet A Collignon A Barc M Bourlioux P Karjalainen T GroEL (Hsp60) of *Clostridium difficile* is involved in cell adherence Microbiol 2001 147 87 96 10.1099/00221287-147-1-87 11160803

[B61] Sullivan L Benett GN Proteome analysis and comparison of *Clostridium acetobutylicum* ATTC 824 and SpoOA strain variants J Ind Biotechnol 2006 33 298 308 10.1007/s10295-005-0050-7 16308714

[B62] Dürre P Hollergschwandner C Initiation of endospore formation in *Clostridium acetobutylicum* Anaerobe 2004 10 69 74 10.1016/j.anaerobe.2003.11.001 16701502

